# *In situ* transmission electron microscopy characterization and manipulation of the morphology, composition and phase evolution of nanomaterials under microenvironmental conditions

**DOI:** 10.1039/d5sc01214g

**Published:** 2025-05-07

**Authors:** Na Li, Xinyang Li, Tian Wang, Bo Wen, Zicheng Yin, Jie Feng, Shengchun Yang, Yawei Yang, Guorui Yang, Shujiang Ding

**Affiliations:** a School of Chemistry, Engineering Research Center of Energy Storage Materials and Devices of Ministry of Education, National Innovation Platform (Center) for Industry-Education Integration of Energy Storage Technology, Xi'an Jiaotong University No. 28, West Xianning Road Xi'an 710049 P. R. China dingsj@mail.xjtu.edu.cn; b MOE Key Laboratory for Non-equilibrium Synthesis and Modulation of Condensed Matter, Key Laboratory of Shaanxi for Advanced Materials and Mesoscopic Physics, State Key Laboratory for Mechanical Behavior of Materials, School of Physics, Xi'an Jiaotong University No. 28 West Xianning Road Xi'an 710049 China ysch1209@mail.xjtu.edu.cn; c Electronic Materials Research Laboratory, Key Laboratory of the Ministry of Education, International Center for Dielectric Research, Shaanxi Engineering Research Center of Advanced Energy Materials and Devices, School of Electronic Science and Engineering, Xi'an Jiaotong University Xi'an 710049 PR China ywyang@xjtu.edu.cn

## Abstract

Nanomaterials possess a broad range of applications in areas such as catalysis, energy, and biomedicine because of their unique properties. However, from the perspective of materials synthesis, there are numerous challenges in the controllable preparation of nanomaterials. These include the control of their size, morphology, crystal structure, and surface properties, which are essential for their performance in specific applications. The fundamental cause of these issues is the limitation in the real-time observation of the growth process of nanomaterials. *In situ* transmission electron microscopy (TEM), on the other hand, overcomes the limitations of traditional *in situ* testing techniques. It enables the real-time observation and analysis of the dynamic structural evolution during the growth of nanomaterials at the atomic scale. This contributes to a profound understanding of the nucleation and growth mechanisms of nanomaterials and facilitates the controlled preparation of nanomaterials. This review centers on the utilization of *in situ* TEM to observe and study the complex dynamic processes of zero-, one-, and two-dimensional nanomaterial growth and evolution in different environments (liquid, gas, and solid phases) at the atomic scale. This is of great significance for the design and preparation of nanomaterials with specific properties. The proposed future development of *in situ* TEM, in combination with advanced data analysis and integration with other techniques, holds great potential for the further advancement of nanotechnology and its applications.

## Abstract 2

This review highlights the application of *in situ* transmission electron microscopy (TEM) in studying the morphology, composition, and phase evolution of 0D, 1D, and 2D nanomaterials in solid, liquid, and gas environments.

## Graphical abstract

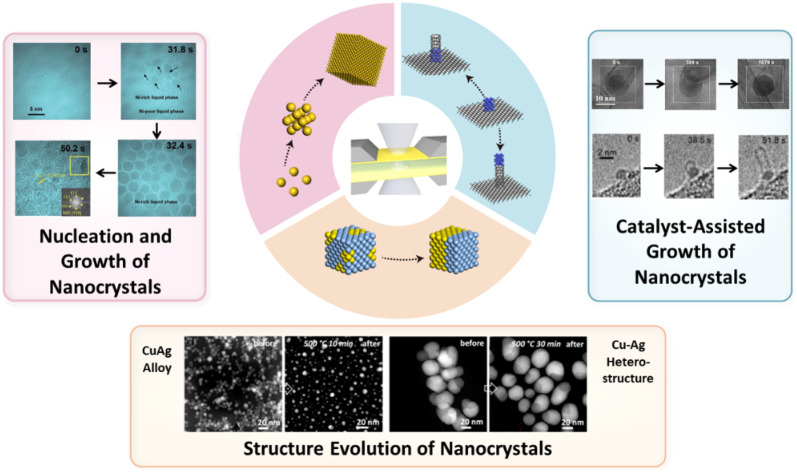

## 1 Introduction

Nanomaterials, defined by their size typically ranging from 1 to 100 nanometers, have ignited a revolution in the field of materials science due to their unique properties and broad spectrum of applications. These materials exhibit distinctive characteristics that are not observed in their bulk counterparts, primarily due to their high surface-to-volume ratios, quantum confinement effects, and the ability to manipulate their structures at the atomic level. Nanomaterials have extremely extensive application prospects in the domains of catalysis,^1,2^ energy,^3^ and biomedicine.^4,5^ Despite their immense potential, the development and application of nanomaterials also present challenges. These include issues related to their synthesis, stability, toxicity, and environmental impact. The controlled fabrication of nanomaterials, or the precise control over their size, shape, crystal structure, and surface properties, is a complex and challenging process that is critical for tailoring their behaviors and optimizing their performance in various applications.^6–8^ The quest for precision in crafting nanomaterials with tailored properties is hindered by a complex interplay of factors that govern their formation, growth, and resultant characteristics. One of the primary obstacles in the controlled synthesis of nanomaterials is the deep understanding and manipulation of nucleation and growth mechanisms at the atomic scale.^9,10^ As demonstrated in the accompanying documents, the journey from monomers to stable nanocrystals is fraught with complexity. Classical and non-classical nucleation theories attempt to explain the aggregation of intermediates into crystalline structures, although the reality of atomic migration dynamics, interfacial evolution, and structural transformation during synthesis often deviates from these theoretical predictions.^11,12^ The uniformity and scalability of nanomaterial synthesis are also significant challenges. The high surface-to-volume ratio of nanomaterials, which is a source of their unique properties, also introduces variability in their synthesis. Techniques such as wet chemical synthesis and solid-state reactions often yield nanomaterials with a broad size distribution and morphological diversity, which can compromise their performance in specific applications. Moreover, the influence of environmental factors during synthesis, such as temperature, pressure, and the presence of surfactants or solvents, adds another layer of complexity. These factors can significantly affect the crystallinity, phase, and surface properties of nanomaterials, leading to a lack of reproducibility in their synthesis. The stability of nanomaterials during synthesis and under various conditions is a critical concern. Phase transformations and structural changes under different stimuli, such as thermal, mechanical, or chemical influences, can alter the intended properties of nanomaterials. For instance, the thermal stability of magnetic nanoparticles^13^ is crucial for their application in high-temperature environments, and any phase change could render them ineffective.^14,15^ In conclusion, the controlled synthesis of nanomaterials is a multifaceted challenge that requires a profound understanding of atomic-scale processes, mastery over environmental influences, ensuring stability and safety, and the development of scalable and environmentally friendly methods. Overcoming these challenges is crucial for the advancement of nanotechnology and the realization of its vast potential in diverse fields of application.

In the quest to unravel the intricate processes underlying the formation and evolution of nanomaterials, *in situ* transmission electron microscopy (TEM) has emerged as a transformative tool, providing a platform for real-time observation and manipulation of nanostructures with atomic accuracy.^16^ The journey of *in situ* TEM began with the desire to transcend the limitations of traditional *ex situ* characterization techniques, which often fell short in capturing the dynamic nature of material synthesis and phase transformations. *In situ* TEM has evolved to fill this void, offering a suite of capabilities that allow researchers to peer into the heart of nanomaterial formation processes. With its ability to operate under a variety of conditions, including high temperatures, pressures, and in the presence of various chemical environments,^17^*in situ* TEM has become an indispensable ally in the quest to understand and control material properties at the fundamental level.^18,19^ The importance of *in situ* TEM in nanomaterial preparation cannot be overstated. It has facilitated the visualization of nucleation events, the tracking of growth pathways, and the exploration of structural dynamics in real time.^20^ This has been particularly crucial in advancing our understanding of phenomena such as Ostwald ripening, phase separation, and defect evolution, which are pivotal in determining the final properties of nanomaterials.^21–23^ Furthermore, *in situ* TEM has been instrumental in elucidating the mechanisms of size-dependent phase transformations and the role of surface energy in stabilizing metastable phases. One of the key strengths of *in situ* TEM lies in its multimodal approach, which integrates imaging with spectroscopic techniques such as energy dispersive X-ray spectroscopy (EDS) and electron energy loss spectroscopy (EELS). This synergy allows for comprehensive characterization of nanomaterials, capturing not only their morphology but also their chemical composition and electronic structure. The implementation of aberration-corrected lenses and the development of advanced imaging modalities, such as high-angle annular dark field (HAADF), scanning TEM (STEM) and electron tomography, have further enhanced the spatial resolution and analytical prowess of *in situ* TEM. The impact of *in situ* TEM extends beyond academic research. It has played a pivotal role in the development of new materials for energy storage, catalysis, electronics, and medicine. For instance, in the realm of catalysis, *in situ* TEM has been used to study the active sites of nanoparticles under reaction conditions, providing insights into their catalytic mechanisms and enabling the design of more efficient catalysts.^24,25^ Similarly, in the field of electronics, the technique has been harnessed to investigate the conduction properties of nanomaterials and their response to electrical stimuli, which is vital for the development of nanoscale devices.^26,27^ As we stand on the precipice of new discoveries, the future of *in situ* TEM holds promise for even greater advancements. The integration of machine learning algorithms and artificial intelligence is set to enhance data analysis and automate the identification of complex structural transformations. Moreover, the ongoing miniaturization of TEM components and the development of more sensitive detectors will further push the limits of spatial and temporal resolution, enabling the capture of fleeting events and transient states in nanomaterial systems. The development of *in situ* TEM has been a monumental achievement in the field of nanoscience, providing a powerful means to study and control the synthesis of nanomaterials. Its ability to offer insights into the atomic-scale processes that govern material properties has not only enriched our fundamental understanding but also paved the way for innovative applications across diverse industries.^28^ As we continue to push the boundaries of this technology, the potential for transformative breakthroughs in material design and engineering remains limitless.

**Fig. 1:**
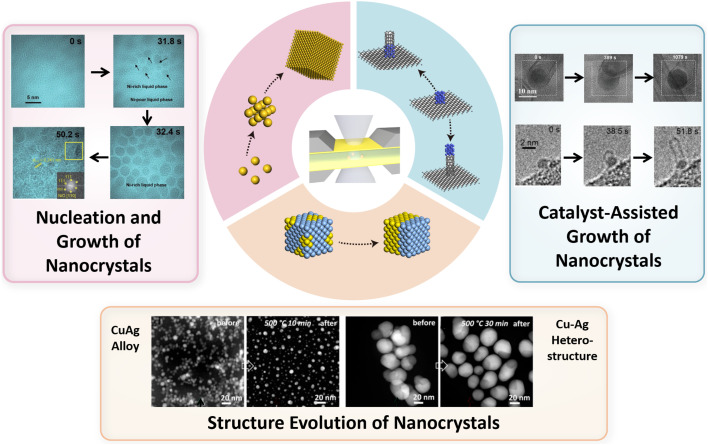
Schematic illustration of key topics in this review. Reproduced with permission.^29–32^ Copyright 2022, Tsinghua University Press. Copyright 2008, American Chemical Society. Copyright 2020, American Chemical Society. Copyright 2019, American Chemical Society.

In this review, we focused on the application of *in situ* TEM in analyzing the morphology, composition, and phase evolution of nanomaterials under microenvironmental conditions (Fig. 1). It highlights the challenges in controlling the synthesis of nanomaterials, such as nucleation and growth mechanisms, environmental influences, and stability issues. *In situ* TEM is presented as a transformative tool that allows real-time observation and manipulation of nanostructures with atomic precision, overcoming the limitations of traditional *ex situ* techniques. The review categorizes *in situ* TEM methodologies into several types, including heating chips, gas-phase cells, and liquid cells, each serving specific roles in nanomaterial synthesis. It discusses the insights gained from *in situ* TEM in understanding the nucleation and growth of nanocrystals, the formation of 0D, 1D, and 2D nanomaterials, and the effects of electron-beam interactions. The article also addresses the challenges associated with *in situ* TEM, such as replicating realistic synthesis conditions, achieving high temporal and spatial resolution, managing electron beam interactions, and integrating with other analytical techniques. Despite these challenges, the review emphasizes the significant contributions of *in situ* TEM to advancing our understanding of nanomaterial synthesis and its potential for future breakthroughs in material design and engineering.

## 2 Classifications of *in situ* TEM for nanomaterials synthesis

**Fig. 2:**
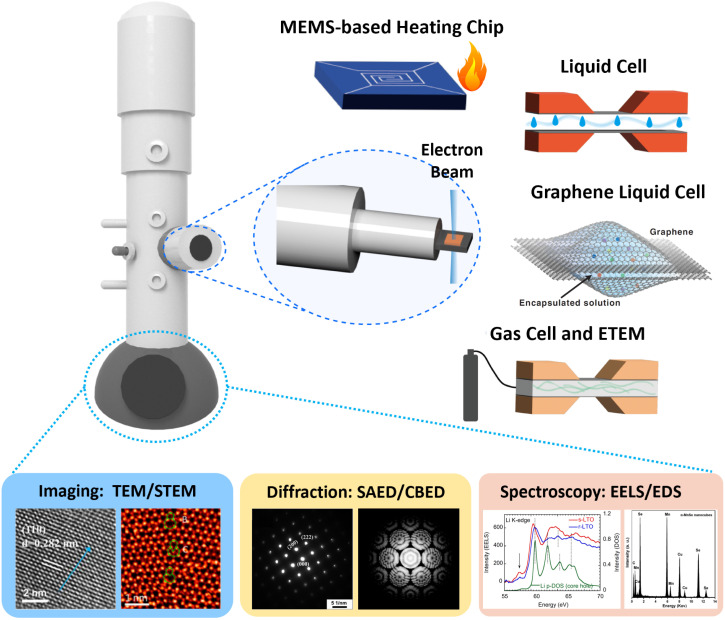
Schematics of TEM and *in situ* chips and cells fabricated by MEMS. Reproduced with permission.^43–46^ Copyright 2013, Elsevier. Copyright 2024, Elsevier. Copyright 2016, American Chemical Society. Copyright 2019, American Chemical Society.

*In situ* TEM methodologies possess the capability to monitor the developmental stages of a particular system through the establishment and activation of its external conditions. Integral to *in situ* TEM are the application of external triggers and the ability to perform real-time monitoring. The former is facilitated by a range of specialized TEM holders, while the latter benefits from the implementation of advanced, rapid recording systems, which are not the focus of this review. To date, the exploration of TEM holders for nanomaterial synthesis has led to the identification of five types: the *in situ* heating chip, the electrochemical liquid cell,^33–35^ the graphene liquid cell,^36–38^ the gas-phase cell,^39,40^ and the environmental TEM,^41,42^ each playing a significant role in this field (Fig. 2).

### 2.1 *In situ* TEM thermal engineering—heating chip

The “heating chip” is a specialized tool utilized in the field of materials science, particularly for the *in situ* study of nanomaterials. It allows for the precise control and application of heat to samples within a TEM, enabling researchers to observe the dynamic evolution of the material structure and chemistry under elevated temperatures. Classifications of heating chips typically focus on the type of heating element and the method of temperature control.^47–49^ For instance, there are furnace-type heating holders, which use a resistive heating element to heat the entire sample, and microelectromechanical system (MEMS)-based heating holders, which offer localized heating through a nanopatterned metal element.^50^ MEMS-based heating chips are often pre-calibrated for temperature control but may require verification under specific experimental conditions due to probable electron-beam-induced heating effects.

In the synthesis of nanomaterials, heating chips play a crucial role by facilitating the study of thermally induced transformations. They allow researchers to monitor processes such as phase transitions, crystal growth, and structural changes in real time. The localized heating provided by MEMS-based chips is particularly beneficial for studying nanostructured and focused ion beam samples, as it minimizes thermal drift and enables rapid temperature stabilization. Moreover, heating chips can be integrated with other *in situ* TEM techniques, such as gas or liquid environments, to simulate realistic conditions for the material under study. This integration provides a comprehensive understanding of the material's behavior under various stimuli, which is vital for the rational design and optimization of nanomaterials for energy-related applications. Heating chips are indispensable for *in situ* TEM studies, providing a controlled thermal environment to observe and analyze the structural and chemical evolution of nanomaterials, thereby contributing significantly to the advancement of materials science and technology.^51^

### 2.2 *In situ* TEM gas environment study—gas cell and environmental TEM

*In situ* TEM for gas environments is primarily classified into two types: environmental TEM (ETEM)^41^ and windowed holders.^18^ ETEMs feature advanced pumping systems that maintain a high vacuum in the microscope column while allowing gas to be introduced around the sample, thus simulating realistic reaction conditions. They have progressed from early models with limited resolution to modern versions capable of atomic-scale imaging. Windowed holders (gas cell), in contrast, are designed to contain both the material and gas within a sealed reactor that can be heated, enabling studies under controlled gas and temperature conditions. These holders are compatible with conventional TEM instruments and allow for experiments under high pressures, making them versatile for various catalytic studies.^52^ In the context of nanomaterial synthesis, *in situ* TEM plays a vital role. It has been used to monitor the transformation of metal particles into single atoms, a process that significantly enhances catalytic efficiency. For example, the evolution of subnanometric metal species within specific spatial confinements has been studied under different gas atmospheres, revealing the dynamics of sintering and redispersion. Additionally, *in situ* TEM has been instrumental in visualizing the diffusion of atoms within matrices, leading to the formation of individual atoms and clusters, which is essential for the development of highly dispersed catalysts.

### 2.3 *In situ* TEM liquid environment study—electrochemical liquid cell and graphene liquid cell

Liquid cell technology has revolutionized the field of nanomaterial synthesis by enabling *in situ* observations under TEM. These cells are designed to contain liquid samples within a high-vacuum TEM environment, allowing researchers to directly visualize dynamic processes such as nanoparticle growth, transformation, and motion at the nanoscale. There are primarily two types of liquid cells: microfabricated silicon cells^33,53^ and graphene liquid cells.^38^ Microfabricated silicon cells are made from silicon wafers and feature electron-beam-transparent windows, typically composed of thin Si_3_N_4_ membranes, which allow for high-resolution imaging. These cells can be static or flow-type, with the latter enabling controlled introduction of reactants for reactions requiring precise temporal and spatial control. Graphene liquid cells, on the other hand, use graphene sheets as the window material, offering even higher spatial resolution due to the thinness and electron transparency of graphene.^54^

In the context of nanomaterial synthesis, liquid cells have been instrumental in studying various phenomena. They have been used to observe the nucleation and growth of nanoparticles, including metal nanoparticles, through processes such as electrochemical reduction.^55^ The real-time imaging capabilities of liquid cells have shed light on the mechanisms of nanoparticle formation, revealing insights into the role of prenucleation intermediates and the dynamics of aggregative growth.^56^ Furthermore, liquid cells have facilitated the investigation of nanoparticle transformations, such as galvanic replacement reactions and etching processes, which are crucial for synthesizing nanoparticles with complex structures and desired properties. The ability to control the chemical environment within liquid cells has also been vital for studying the effects of various factors on nanoparticle synthesis, such as the concentration of precursors, the presence of stabilizing agents, and the influence of the electron beam itself.^17^ By manipulating these parameters, researchers can gain a deeper understanding of the underlying chemistry and optimize the synthesis of nanomaterials for various applications. Liquid cell technology has expanded the horizons of nanomaterial research by providing a platform for *in situ* TEM studies, offering unprecedented insights into the formation, transformation, and behavior of nanoparticles in liquid media. This technology has become an indispensable tool for scientists in the fields of materials science, chemistry, and nanotechnology.

## 3 Nucleation and growth of nanocrystals

### 3.1 Nucleation of nanocrystals

Over the past few years, there has been a consistent rise in the utilization of functional nanocrystals across different sectors, with a notable increase in energy, catalysis, and biomedicine applications.^1,3^ This surge is primarily attributed to the distinctive structural characteristics of nanocrystals, such as their extensive specific surface area, one of a kind surface, and electron configurations, which hold promise for these applications. Nonetheless, crafting nanoparticles with the desired structures and attributes poses considerable difficulties. To advance further, a variety of theories regarding the nucleation and development of nanocrystals have been put forward, encompassing both classical and non-classical nucleation concepts, as well as growth mechanisms like ripening, clustering, and merging.^12^ Despite these theoretical advancements, the underlying atomic movements, regulatory elements, and propelling forces of these phenomena are not yet fully understood.^21^ In this context, the use of *in situ* TEM is emerging as an indispensable instrument for scientific inquiry, enhancing our comprehension of the mechanisms behind nanocrystal development. The insights gained from these studies are instrumental in the deliberate alteration of the structure and form of nanocrystals, ultimately guiding the production of high-caliber nanocrystals tailored for diverse applications.

**Fig. 3:**
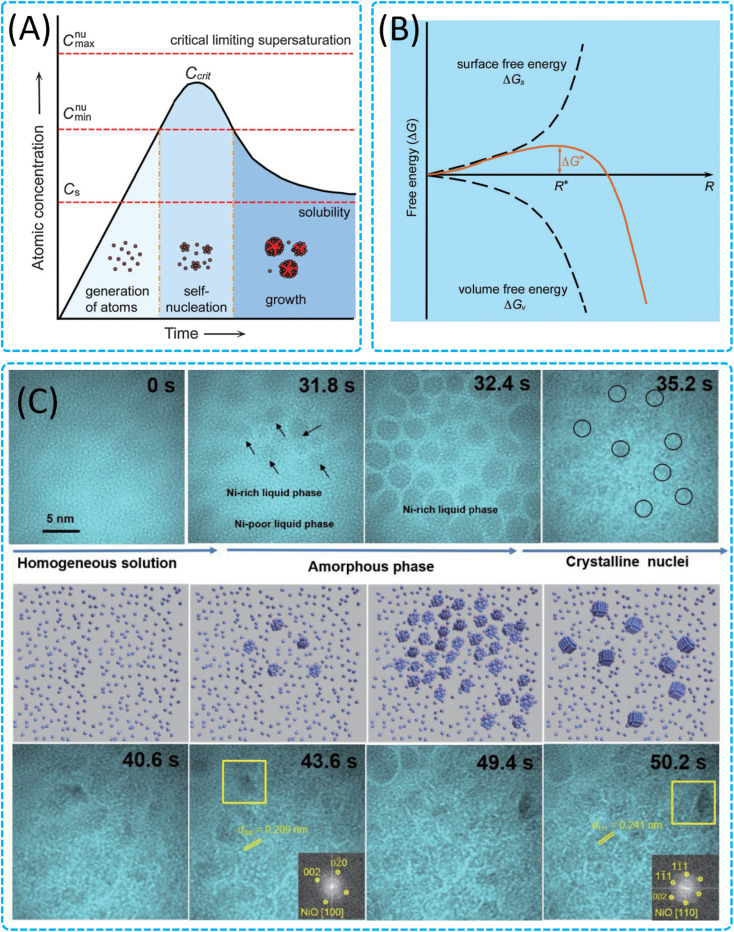
Nucleation of nanocrystals. (A) LaMer model describing nucleation and growth of nanocrystals as a function of reaction time and concentration of precursor atoms. Reproduced with permission.^58^ Copyright 1950, American Chemical Society. (B) Classical nucleation model showing the free energy diagram for nucleation. Reproduced with permission.^59^ Copyright 2009, American Chemical Society. (C) TEM images showing the formation of NiO nanocrystals in SiN_*x*_ liquid cells. Reproduced with permission.^29^ Copyright 2022, Tsinghua University Press.

Classical nucleation theory is a fundamental concept in understanding the formation of nanocrystals.^57^ The classical LaMer model (Fig. 3A)^58^ is an extension of classical nucleation theory. It describes the process by which a new thermodynamic phase forms through the aggregation of monomer units. This theory outlines the process by which nanoparticles nucleate and grow in a solution phase. Here is a summary of the key points related to classical nucleation theory:

#### 3.1.1 Nucleation process

The nucleation of nanoparticles is described as a two-step process involving the formation of stable nuclei from supersaturated precursor species, followed by their growth into larger particles. The nucleation event is considered a critical phase transition from a supersaturated solution to a solid phase.

#### 3.1.2 Thermodynamic driving force

The spontaneous phase transition is driven by the exothermicity of lattice formation. The free energy change (Δ*G*) for nucleation is determined by the sum of the phase transformation (Δ*G*_v_) and the solid surface formation (Δ*G*_s_). The negative Δ*G*_v_ contributes to the system's overall stability, while the positive Δ*G*_s_ increases with the surface area of the nuclei (Fig. 3B).^59^

#### 3.1.3 Critical nucleus size

There is a critical size (R*) at which the total free energy change (ΔG*) reaches a maximum. Nuclei smaller than this critical size are unstable and tend to dissolve, while larger nuclei are more stable and can grow.

#### 3.1.4 Kinetics of nucleation

The nucleation rate is influenced by the mobility of precursor species and the temperature of the reaction. The Arrhenius equation is used to describe the temperature dependence of the nucleation rate, where an increase in temperature can significantly affect the rate due to the increased mobility of precursors.

#### 3.1.5 Supersaturation

Classical nucleation theory emphasizes the importance of supersaturation for nucleation to occur. A certain concentration of nanocrystals, known as the critical concentration (*C*_crit_), must be reached for nucleation to take place.

#### 3.1.6 Energy barrier

There is an energy barrier associated with nucleation, which must be overcome for the process to proceed. This barrier is related to the maximum free energy change (ΔG*) that occurs at the critical nucleus size.

#### 3.1.7 Growth of nuclei

Once stable nuclei are formed, they can grow at a lower concentration of precursor species that is slightly above the saturation concentration (*C*_s_). This growth process is less energy-consuming compared to the nucleation process.

#### 3.1.8 Control of nucleation

Classical nucleation theory suggests that controlling the nucleation process can be achieved by modulating the surface free energy and/or volume free energy, which in turn affects the total free energy's dependence on the size of the nuclei. This can be done by varying surfactants, forming nuclei of different materials that can transform into the desired nuclei, or changing the reaction environment.

#### 3.1.9 Reversible disorder–order transitions in atomic crystal nucleation

Atomic crystal nucleation, a critical process in materials science, has long been misunderstood. Recent advances in classical nucleation theory have revealed that the nucleation process is more complex than previously thought, involving non-classical mechanisms such as dynamic and reversible structural fluctuations between disordered and crystalline states. *In situ* TEM observations of gold nanocrystal nucleation on graphene surfaces, with millisecond temporal resolution, have shown that the early stages of atomic crystallization are characterized by repeated transformations between these two states.^60^ This dynamic process challenges the traditional view of nucleation as a single irreversible transition and highlights the role of metastable states and atomic-scale dynamics in determining the nucleation pathway. The study found that small atomic clusters exhibit a high probability of retaining a disordered state, with the relative population of crystalline states increasing as the cluster grows. Once a certain size is reached, the cluster becomes trapped in the crystalline state due to increased energy differences. This size-dependent thermodynamic stability is attributed to the low energy barrier for crystalline-to-disordered transitions in small nanoclusters, which can be overcome by various energy sources such as monomer attachment and electron beam interactions. These findings not only improve our understanding of the fundamental mechanisms underlying material growth processes such as thin-film deposition, interface-induced precipitation, and nanoparticle formation but also provide insights into the atomic-scale processes that govern material properties.

Classical nucleation theory provides a framework for understanding and controlling the initial stages of nanoparticle formation, which is crucial for the synthesis of colloidal nanoparticles with tailored properties (Fig. 3C).^29^

### 3.2 Growth of 0D nanomaterials

The realm of zero-dimensional (0D) nanomaterials has witnessed remarkable advancements in both applications and fabrication techniques, marking a significant evolution in nanotechnology.^61^ 0D nanomaterials, with their distinct properties arising from quantum confinement effects and large surface-to-volume ratios, have catalyzed a surge of interest across various fields, which encompass energy conversion,^62,63^ catalysis,^64,65^ photonics,^66^ and biology.^67,68^ For instance, in the energy sector, these nanomaterials have been utilized in solar cells^69–71^ and batteries,^72–74^ capitalizing on their size-dependent electronic properties. In catalysis,^75^ 0D nanomaterials have demonstrated enhanced activity and selectivity for various chemical reactions, attributed to their well-defined surface structures and compositions. The photonics industry has also benefited from the unique optical properties of 0D nanomaterials, such as in light-emitting diodes and photodetectors.^76,77^ Moreover, in the biomedical field,^78^ their use in drug delivery systems and imaging agents has shown great promise. The synthesis of 0D nanomaterials has been refined through various approaches, ensuring control over size, shape, and composition–crucial parameters that dictate their properties and performance. Wet chemical methods, including sol–gel, precipitation, and hydrothermal synthesis, have been refined to produce monodisperse 0D nanomaterials with high yields.^15,79,80^ Advanced physical techniques such as sputtering and laser ablation have also been employed to synthesize nanomaterials with unique properties.

Furthermore, the integration of *in situ* TEM allows researchers to directly visualize the dynamic processes of nanomaterial synthesis, including nucleation, growth, and phase transformations, which are crucial for understanding the underlying mechanisms and optimizing the material properties.^56,81,82^ The ability to apply various external stimuli, such as mechanical, thermal, electrical, and chemical influences, within the TEM environment provides a unique platform for the precise control and tuning of the nanomaterial properties. Advancements in *in situ* TEM techniques have led to the development of new experimental setups that enable the study of 0D nanomaterials under more realistic and complex conditions. These include the use of nanomanipulation for mechanical testing, MEMS for precise temperature control, and environmental cells for gas and liquid exposure.^23,52,53,83^ Furthermore, the integration of spectroscopic tools like EELS and EDS within the TEM has enhanced the chemical and electronic characterization capabilities. Looking forward, the direction of research in *in situ* TEM for 0D nanomaterials is expected to focus on achieving higher spatial and temporal resolutions, improving the stability and controllability of external stimuli, and expanding the range of accessible materials and conditions. The development of novel *in situ* holders and the integration of additional physical fields, such as magnetic and optical stimuli, will further broaden the applicability of *in situ* TEM in the synthesis and study of 0D nanomaterials. Moreover, the combination of *in situ* TEM with computational modeling and simulation will provide deeper insights into atomic-scale processes and enable the prediction and design of new materials with tailored properties.

#### 3.2.1 Growth of 0D nanomaterials through solid-state interactions

In the field of materials science and nanotechnology, a thorough understanding of the control of nanoparticle size and morphology during high-temperature solid-phase synthesis, structural changes, phase transitions and the stability of surface composition and morphology is essential.^84,85^ These factors significantly affect the performance and long-term stability of materials in applications such as fuel cell catalysis. In this paper, we comprehensively discuss a number of key factors affecting the structure and performance of materials at high temperatures, including grain growth,^85–88^ thermal stress-induced phase transitions,^89,90^ agglomeration or sintering of nanoparticles,^47,91^ diffusion processes in solid phase reactions,^92–94^ compositional inhomogeneities in multicomponent systems,^95,96^ oxidation and impurity introduction at elevated temperatures,^97,98^ and phase transition^99^ issues detrimental to late-stage catalytic reactions. The *in situ* heating techniques was used to observe structural changes in nanoparticles, in particular the study of performing *in situ* heating of PtNi_1.5_ octahedral nanoparticles within TEM to study their compositional and morphological changes.^98^ These studies provide new insights into the factors influencing catalyst activity and stability and reveal the role of metal clusters in catalysis and crystal nucleation. In addition, Xia *et al.* documented the synthesis of Ru octahedral nanocrystals, thermal stability at elevated temperatures, migration and morphological evolution of metal clusters.^100^ Gatalo *et al.* investigated PtCu_3_/C and PtM-based alloy nanoparticles as oxygen reduction reaction (ORR) electrocatalysts, providing a significant scientific foundation for the design and optimization of ORR electrocatalysts in fuel cells.^101^ Furthermore, the exploration and study of the thermal stability of gold (Au) nanoparticles,^88^ the thermal evolution of C–Fe–Bi nanocomposite^92^ systems and the dynamic changes of Pt–Co nanoparticles^102,103^ during heat treatment provide an important theoretical basis for understanding and controlling the microstructural evolution of materials. The results of these studies are important for the development of new carbon-based materials, optimization of catalyst properties and understanding the formation mechanism of intermetallic compound nanoparticles.

**Fig. 4:**
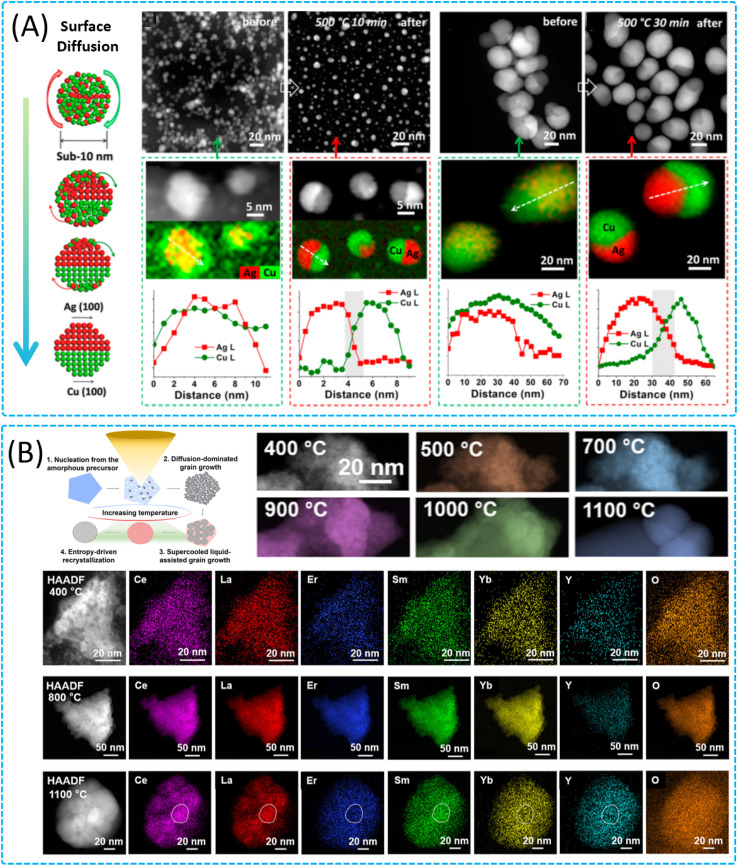
Phase transition of 0D nanomaterials in solid-state interactions. (A) Schematic representations of the reasonable atomic motion in sub-10 nm CuAg nanoparticles during annealing to form a Cu (100)/Ag (100) heterointerface, and STEM-HAADF images of CuAg nanoparticles before and after annealing under vacuum at 500 °C for 10 min. The corresponding high-magnification STEM-HAADF images and EDS elemental maps. Reproduced with permission.^32^ Copyright 2019, American Chemical Society. (B) Schematic representations of the HEFO from an amorphous precursor and characterization of the structures and compositions of high-entropy fluorite oxides at different temperatures. Reproduced with permission.^97^ Copyright 2022, American Chemical Society.

Meanwhile, studies on the thermal stability and growth dynamics of the structures of novel functional nanoparticles (bimetallic Janus nanostructures (JNs)^32,104–106^ and high-entropy oxides (HEOs)^97^) have extended our understanding of the evolutionary behavior of nanoparticles at elevated temperatures and provided important scientific insights into the control of the nanoparticle structure and morphology. These findings not only deepen our understanding of the behavior of nanoparticles at high temperatures, but also provide an important scientific basis for the design of materials with specific properties, especially for applications in catalysts, energy storage, and conversion. JNs have garnered significant attention due to their distinctive interfacial properties that can be finely tuned for a myriad of applications. The size of these nanoparticles plays a pivotal role in dictating the structural and orientational characteristics of their heterointerfaces, which in turn significantly influences their performance in practical applications. Sun *et al.* utilized *in situ* annealing HRTEM and revealed a novel sub-10 nm heterostructure with a unique interface, which offered fresh insights into the role of particle size in interfacial evolution during thermal annealing (Fig. 4A).^32^ The study underscores the importance of understanding the atomic motion mechanism that governs the formation of different heterointerfaces, influenced by particle size. The findings are instrumental in the development of nanoelectronic devices and catalytic systems where precise control over the heterointerfaces is essential for optimizing performance. In the realm of materials science, HEOs have emerged as a novel class of multicomponent solid-solution materials, showcasing immense potential for applications in catalysis, energy storage, and thermal barrier coatings. The ability to fine-tune their composition and crystal structures offers a vast landscape for enhancing material properties. However, unraveling the atomic-scale mechanisms of nucleation and growth of HEOs has been a formidable challenge, impeding the rational design of their structure and function. Gao *et al.* leveraged atomic resolution *in situ* STEM to visualize the entire formation process of a high-entropy fluorite oxide (HEFO) from a polymeric precursor, as schematically shown in Fig. 4B.^97^ The findings of this study are pivotal, providing critical insights into the rational synthesis of HEOs with controlled grain sizes and morphologies, which in turn are essential for tailoring the material's properties. The research demonstrates that the random and uniform distribution of elements in the designed polymeric precursor is fundamental to the low-temperature oxidation and nucleation process. Furthermore, the study elucidates that the formation of HEFO entails slow grain growth through atom diffusion at temperatures below 900 °C and a subsequent liquid-phase-assisted grain growth process at elevated temperatures. This work not only advances the scientific understanding of HEOs but also opens new avenues for the development of advanced materials with targeted properties for specific technological applications.

#### 3.2.2 Growth of 0D nanomaterials in the liquid phase

Liquid-phase preparation of nanoparticles allows precise regulation of functional materials at the molecular level, which is crucial for the realization of “atom-to-atom” design and construction of materials. The growth mechanism of nanoparticles in the liquid phase is a complex and delicate dynamic process that involves multiple stages, including nucleation,^107^ growth,^108^ and ultimately the maintenance of particle stability.^29,109^ Currently, the growth mechanisms of nanoparticles in the liquid phase mainly include the surface reaction mechanism,^81,110^ monomer diffusion mechanism,^111^ LaMer theory,^9,58^ Ostwald ripening and digestive ripening,^112,113^ Finke–Watzky mechanism,^114,115^ oriented attachment mechanism,^116–118^ intraparticle growth,^119,120^ core–shell growth^121–125^ and interfacial phase growth.^126^ The growth mechanism of nanoparticles in the liquid phase not only determines the size and morphology of nanoparticles, but also affects their physicochemical properties.

**Fig. 5:**
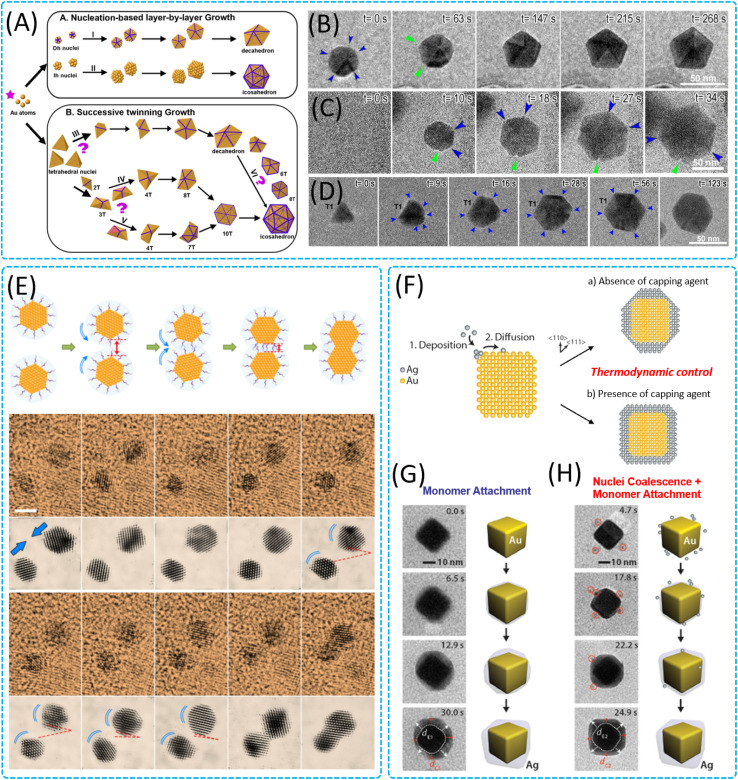
Nucleation, growth and structural transformation of 0D nanomaterials in the liquid phase. (A) Schematic of different formation routes of decahedral and icosahedral multiply twinned nanoparticles. Time-sequenced TEM images of nucleation-based layer-by-layer growth of decahedral and icosahedral Au NPs from the rounded multiply twinned seeds viewed along (B) [011] and (C) [111] orientations, respectively. (D) Time-sequenced TEM images of successive twinning growth of an icosahedral Au MTP from an initial tetrahedral seed viewed along [111] orientation. Reproduced with permission.^131^ Copyright 2020, American Chemical Society. (E) Schematic illumination of the whole oriented attachment process and imaging of oriented attachment at the atomic level. Reproduced with permission.^132^ Copyright 2018, Springer Nature. (F) Schematic illustrations showing the shape evolution of a cubic seed under thermodynamic control for two conditions: absence or presence of capping agents. Time-lapse TEM images of Au nanocubes interacting with the Ag precursor solution inside a flow cell *via* (G) the monomer attachment process and (H) monomer attachment and nuclei coalescence. Reproduced with permission.^124^ Copyright 2016, American Chemical Society.

As an important class of nanomaterials, twinned noble metal nanoparticles show a wide range of applications and significant technological advantages in many fields, such as catalysis, optics, electronics, and biomedicine, due to their excellent physicochemical properties and tunable surface plasmon resonance.^127–130^ The growth process of such noble metal nanoparticles in liquids is mostly kinetically controlled, with a large deviation from the equilibrium state defined by thermodynamics. When the temperature increases, various changes in the arrangement of atoms occur, which affect the geometrical morphology of the nanocrystals, the spatial distribution of the elements, the internal structure and the phase structure. Ma *et al.* used an *in situ* LCTEM technique to study in depth multi-twinned gold nanoparticles, especially decahedra and icosahedra, which have a wide range of applications and significant technological advantages (Fig. 5A).^131^ The growth of multi-twinned nanoparticles mainly proceeds through two paths: path A involves core-based laminar growth, starting from rounded multi-twinned seeds and growing in a laminar manner (Fig. 5B and C), and path B involves continuous twinning and tetrahedral (tetrahedra) growth (Fig. 5D). There were differences in the internal strain relaxation mechanisms and growth kinetics of the two pathways: in path A, multi-twinned nanoparticles grew by opening and closing of re-entrant grooves at twin boundaries, which was not found in path B. The researchers discussed the preferred pathway (path A) and the preferred pathway (path B) further. The researchers further discussed the relationship between the preferred path (A or B) and the initial seed yield as well as the size- and morphology-dependent multi-twinned nanoparticle formation, revealing the mechanism of formation and evolution of multi-twinned structures. Zhu *et al.* conducted an *in situ* study of the growth mechanism of citrate-stabilized gold nanoparticles by oriented attachment (Fig. 5E).^132^ It was observed that the process of oriented attachment between pairs of nanoparticles in the liquid phase was tightly controlled by the adsorbed ligand layer, and when two gold nanoparticles approached a certain distance, their ligand layers started to overlap, resulting in the rotation of the pairs of particles shifting from a random mode to an oriented mode until their <111> crystalline surfaces were completely aligned, followed by a rapid contact. The thickness and interaction energy of the ligand layer in this process are the key factors determining the oriented attachment behavior of nanoparticles. For the core–shell growth mechanism, Tan *et al.* thoroughly explored the epitaxial growth process of silver (Ag) on gold nanocubes (Au nanocubes) in solution (Fig. 5F).^124^ It was found that the formation of the Au–Ag core–shell structure proceeds through two mechanisms: the first mechanism is fusion, where silver nanoparticles are adsorbed onto Au nanocubes (Fig. 5G), and the second mechanism is monolithic attachment, where silver atoms are epitaxially deposited on Au nanocubes (Fig. 5H). Both paths end up with the same Au–Ag core–shell nanostructure. Further analysis reveals that the growth of silver shells is controlled kinetically and thermodynamically. When the surface diffusion rate is faster than the atomic deposition rate, the reaction is driven by thermodynamics, and the silver atoms diffuse and migrate to the sites with the lowest surface free energy, the growth of the shell layer will proceed along the <100> and <110> directions. However, when the atomic deposition rate is higher than the surface diffusion rate, the reaction is controlled by kinetics, with preferential attachment of silver atoms to certain crystalline surfaces, and the shell layer growth pattern may shift to promote the formation of more complex shapes, such as concave cubes or octopods; the discovery of these phenomena is of great significance for the understanding and optimization of the synthesis process of core–shell nanostructures.

#### 3.2.3 Growth of 0D nanomaterials through gas–solid interactions

**Fig. 6:**
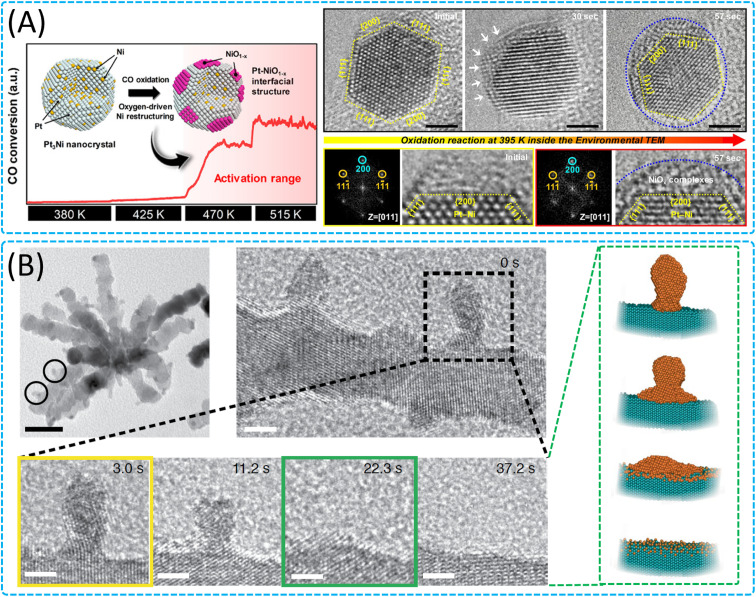
Structural and phase transformation of 0D nanomaterials in gas–solid interaction. (A) Pt–NiO_1−*x*_ interfacial structure formation of a Pt_3_Ni nanoparticle after O_2_ annealing and *in situ* TEM of the spreading process. Reproduced with permission.^145^ Copyright 2020, American Chemical Society. (B) The growing and spreading process of Pt islands on Ru branched nanoparticles to create single-Pt-atom-on-Ru catalysts, and the HRTEM images and corresponding cartoons of the island as it transforms during *in situ* heating under a flow of H_2_. Scale bars, 2.5 nm. Reproduced with permission.^146^ Copyright 2022, Springer Nature.

The complex dynamic behaviour of nanoparticles in gas–solid reactions mainly involves processes such as alloying,^133,134^ segregation,^135^ phase transition,^136,137^ redox reactions,^138–142^ lattice reconstruction^140,143^ and morphological evolution,^141,144^ which together determine the stability, activity and selectivity of the nanoparticles. Kim *et al.* found that when heated in an oxygen atmosphere, the surface of Pt_3_Ni nanoparticles undergoes reconfiguration (Fig. 6A), resulting in the formation of a Pt–NiO_1−*x*_ interfacial structure.^145^ The newly formed Pt–NiO_1−*x*_ interfacial structure plays a key role in the CO oxidation reaction and promotes the improvement of catalytic activity. In addition, with regard to the formation process and catalytic mechanism of noble metal single atoms, which has received much attention, the structural changes of Pt nanoparticles were observed in real time by ETEM by the Tilley group (Fig. 6B).^146^ The thermodynamically driven rearrangement process of Pt islands on low-index Ru crystal surfaces was observed by heating Pt–Ru nanomaterials in an H_2_ atmosphere, and the Pt atoms were gradually and uniformly dispersed on the surface of the Ru nanoparticles as the reaction time progressed, eventually forming discrete individual Pt atoms. This change was driven by the combination of decreasing the surface free energy of Pt islands and increasing the strong bonding interaction between Pt and Ru, which contributed to the final dispersion of Pt atoms on the surface of branched Ru nanoparticles and the formation of Pt single atom catalysts with high activity and resistance to CO poisoning. Meanwhile, the phase separation mechanism of the high-entropy alloy nanoparticles in high-temperature environments under different atmosphere bars was thoroughly investigated by the group of Zachariah. They inspected the high-temperature reduction dynamics of oxidised FeCoNiCuPt high-entropy alloy nanoparticles^147^ in a hydrogen environment and found that the outer surface of the oxide layer of the oxidised FeCoNiCuPt high-entropy alloy was transformed into a porous structure in a H_2_ atmosphere, where the oxidised state Cu was completely reduced to Cu NPs, while Fe, Co and Ni remained in the oxidation state. During this process, the core of the oxidized FeCoNiCuPt high entropy alloy nanoparticles shrinks due to the outward diffusion of the transition metals and their associated vacancies, leading to the formation of a gap between the core and the inner surface of the oxide layer, which expands due to the outward diffusion fluxes of all the transition metals (Fe, Co, Ni, and Cu). At the same time, they thoroughly investigated the phase separation mechanism of Fe_0.28_Co_0.21_Ni_0.20_Cu_0.08_Pt_0.23_ high-entropy alloy nanoparticles^148^ during high-temperature oxidation and found that their oxidation at 400 °C was controlled by the Kirkendall effect. Transition metals (Fe, Co, Ni and Cu) diffuse outwards to form disordered oxide layers, and locally ordered lattices are observed in the oxides, suggesting that Fe_2_O_3_, CoO, NiO and CuO grains are formed in the overall disordered matrix.

### 3.3 Growth of 1D nanomaterials

1D nanomaterials, with their distinct structural and functional attributes, have emerged as a cornerstone in the realm of nanotechnology.^149^ The defining features of 1D nanomaterials include their ultra-high aspect ratios, tunable cross-sectional dimensions ranging from 1 to 100 nanometers, and variable lengths that can span from hundreds of nanometers to millimeters. These attributes endow 1D nanomaterials with a suite of unique properties that are leveraged across a myriad of applications. The high surface-to-volume ratio of 1D nanomaterials facilitates exceptional interaction with their environment, making them ideal candidates for applications in sensing, catalysis, and energy storage. Their quantum confinement effects, stemming from their small cross-sectional dimensions, lead to size-dependent electronic and optical properties, which are pivotal for the development of advanced photonic and electronic devices. Moreover, the crystalline nature of 1D nanomaterials ensures high purity and fewer defects, contributing to their superior performance in various applications. The tunable bandgap and efficient charge transport properties of semiconductor nanowires have been particularly instrumental in the advancement of integrated photonics, energy conversion, and storage technologies.^150,151^ The ability to precisely control the nucleation and growth of these nanomaterials is crucial for tailoring their structures and properties to meet specific application requirements. In this context, *in situ* TEM has emerged as a powerful tool for providing real-time, atomic-scale insights into the formation and evolution of 1D nanomaterials.

*In situ* TEM allows researchers to directly observe the dynamics of nanocrystal nucleation, growth, and structural transformations under controlled conditions, which is essential for understanding the fundamental mechanisms governing their formation. This technique has been particularly instrumental in studying the effects of various reaction parameters, such as temperature, atmosphere, and substrate interactions, on the nucleation and growth behaviors of 1D nanomaterials. By employing *in situ* TEM, researchers have been able to reveal the atomic migration dynamics, interfacial evolution, and phase transformations during the synthesis of nanowires, nanotubes, and other 1D structures. Advancements in *in situ* TEM have also led to the development of new synthetic strategies, such as catalyst-assisted growth mechanisms, including vapor–liquid–solid (VLS)^152–154^ and vapor–solid–solid (VSS)^155–157^ pathways, which have been extensively studied for the controlled fabrication of 1D nanomaterials. These *in situ* studies have provided valuable insights into the role of catalysts, the influence of gas environments, and the mass transfer processes during nanowire growth, enabling the optimization of synthesis conditions for the production of high-quality 1D nanomaterials with desired properties. Moreover, *in situ* TEM has been pivotal in exploring the growth of 1D nanomaterials under various conditions, including the effects of strain, defects, and surface/interface engineering. The technique has also been used to investigate the dynamics of light elemental atoms in metal nanocrystals during catalytic reactions, which is crucial for understanding their catalytic performance and potential applications. Looking forward, the continued development of *in situ* TEM techniques, such as the integration of more complex growth environments and advanced characterization methods, holds great promise for gaining a deeper comprehension of the nucleation and growth processes of 1D nanomaterials.

#### 3.3.1 Growth of 1D nanomaterials through solid-state interactions

**Fig. 7:**
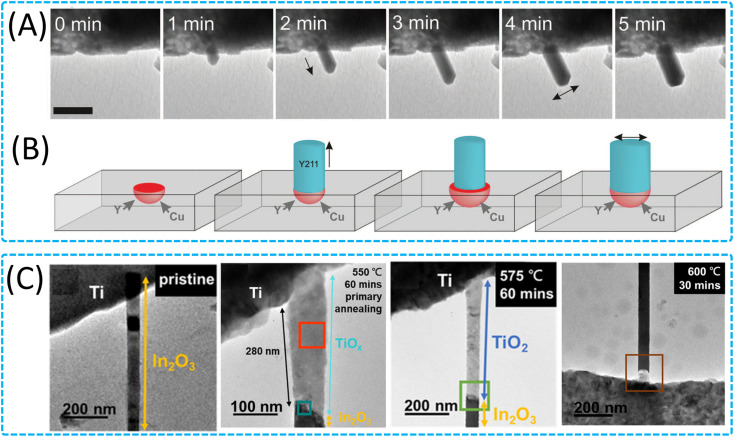
Growth of 1D nanomaterials through solid-state interactions. (A) TEM images and (B) schematic of a nanowire spontaneously broadening as a result of microcrucible creep and expansion on continuing heating at 500 °C. Reproduced with permission.^165^ Copyright 2014, AAAS. (C) Characterization of the pristine In_2_O_3_ nanowire and different temperature annealed In_2_O_3_/TiO_*x*_ heterostructure nanowire. Reproduced with permission.^166^ Copyright 2022, Elsevier.

In the current rapid development of nanoscience and technology, nanowires, which are a type of one-dimensional nanostructure possessing distinctive electrical, optical, and chemical properties, have emerged as a focal point for research and application.^149,150^ They exhibit considerable potential in areas such as microelectronics, sensors, optoelectronic devices, and so on.^151,158,159^ However, precise control of the growth direction, structure and size of nanowires at the atomic level is very complex and requires an in-depth understanding of interatomic interactions and dynamical processes. Especially in solid-state non-catalytic reaction processes, the origin and migration mechanisms of atoms may be different from catalytic growth, and more theoretical studies are needed to reveal these mechanisms. Using *in situ* heating TEM, researchers have carried out detailed studies on different types of nanowire growth mechanisms, covering a wide range of aspects such as the growth of metal oxide nanowires,^160^ the growth of non-catalytic ZnO nanopillars^161^ and Ag_2_S-catalysed ZnS nanowires,^162^ the in-plane growth of silicon nanowires (Si NWs) with indium (In) as a catalyst,^163^ and the evolution of the anisotropy of InAs nanowires^164^ and Au nanowires.^48^ These studies not only provide an in-depth understanding of the kinetics and physicochemical processes of nanowire growth, but also directly observe the dynamic processes of nanowire growth by *in situ* TEM, revealing key mechanisms such as the microcrucible mechanism, the gas–liquid–solid (VLS) mechanism, and the formation of heterostructure nanowires. Boston *et al.* not only directly observed the nanowire growth process, but also revealed a novel microcrucible mechanism, which is a unique way to promote the growth of metal oxide nanowires at high temperatures, opening up a new chapter in the growth of metal oxide nanowires (Fig. 7A and B).^165^ Through a precise experimental design, the team tracked the growth of tetrameric metal oxide nanowires (Y_2_BaCuO_5_) and discovered the melting and diffusion behaviours of barium carbonate (BaCO_3_) nanoparticles during heating, which form droplets on the surface of the porous substrate that act as catalytic points, triggering the growth of Y_2_BaCuO_5_ nanowires. The key features of this microcrucible mechanism include the melting and diffusion of nanoparticles, formation of droplets as a microcrucible and catalytic points, growth of nanowires, maintenance of a fixed diameter growth, dynamics of the liquid–solid interface, and diversity of nanowire morphology. These steps work together to make the nanowire growth process a dynamic and complex process involving interactions between the droplet and the solid matrix. The understanding and application of the microcrucible mechanism provide new strategies for controlling the growth conditions, morphology, and properties of complex oxide nanowires. Chang *et al.* controlled the crystallinity and microstructure of TiO_2_/In_2_O_3_ nanowires through solid-state reactions and observed the In_2_O_3_/TiO_2_ heterostructured nanowire formation process in a high vacuum environment with *in situ* TEM (Fig. 7C).^166^ It was found that at the beginning of the reaction, Ti atoms diffused into the In_2_O_3_ nanowires along the [100] direction to form amorphous TiO_*x*_, and with the annealing process, TiO_*x*_ gradually transformed into polycrystalline TiO_2_, indicating that the secondary annealing could improve the crystallinity of the material, and the study revealed the effect of different annealing temperatures on the formation of heterostructures of the nanowires and the photoresponse properties of these heterostructures in the enhancement. These *in situ* observations on the growth mechanism of nanowires not only advance the theoretical understanding of nanowire growth, but also provide the scientific basis and technical support for future applications of nanowires in electronics, photonics and other high-tech fields.

#### 3.3.2 Growth of 1D nanomaterials in the liquid phase

**Fig. 8:**
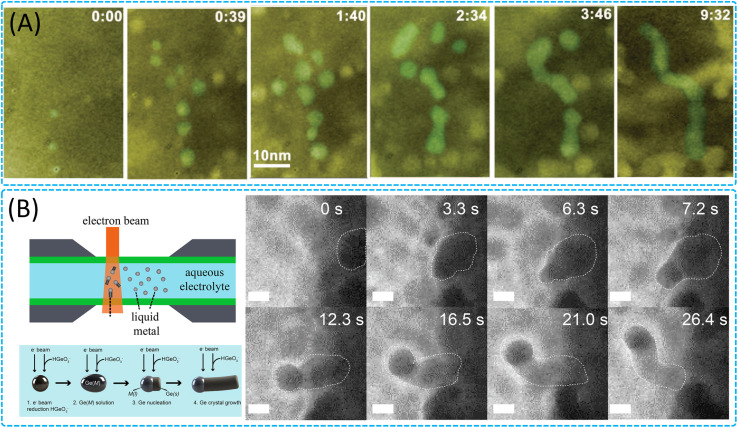
Growth of 1D nanomaterials in the liquid phase. (A) Sequential color TEM images showing the evolution from the initial nucleation and growth in the molecular precursor solution to a later stage of nanowire formation by shape-directed nanoparticle attachment. Reproduced with permission.^176^ Copyright 2012, AAAS. (B) Schematic depiction of Ge nanowire growth and frame grabs with a Ga nanodroplet immersed in an aqueous solution. Reproduced with permission.^55^ Copyright 2020, American Chemical Society.

The liquid environment provides a mild and controllable growth medium, enabling the growth of nanowires at lower temperatures, and the structure, size, shape, and composition of the nanowires can be precisely regulated by adjusting the conditions of solution components, concentration, reaction temperature, and time.^167–169^ In liquid-phase reactions, the study of nanowire growth mechanisms helps to deeply understand the physicochemical processes at the nanoscale, explore new synthesis methods and growth mechanisms, and achieve structural control and performance optimisation of nanowires.^170–173^ For example, studies on the growth mechanism of catalyst-free nanowires have rarely been reported, so the growth kinetics of catalyst-free nanowires are still unknown.^174^ Asghar *et al.* revealed the microscopic growth mechanism of ceria nanowires through the study of *in situ* TEM.^175^ Meanwhile, Liao *et al.* revealed the catalyst-free growth of Pt_3_Fe nanorods with a noble metal alloy-like material in solution (Fig. 8A).^176^ It was found that the growth of nanorods went through three distinct stages: initial nucleation, formation of nanoparticle chains and shaping of nanorods. The Pt and Fe precursors were reduced by electron beam irradiation to form many small nanoparticles, which grew either by monolithic attachment or by merging. The nanoparticles interacted with each other through shape-directed nanoparticle attachment to form nanoparticle chains. The chains were initially curved and polycrystalline but as growth proceeds, they straighten through structural relaxation and eventually form single-crystal nanorods. It was observed that individual nanoparticles or short chains can serve as the basic building blocks for the formation of Pt_3_Fe nanowires. During the formation of nanoparticle chains, the relative positions and orientations between nanoparticles change over time, suggesting that the nanoparticles in the chains can undergo relative motion and orientation changes. During the final growth phase, neighboring nanoparticles in the chain come into contact and form a neck, which is subsequently eliminated by mass redistribution to form a smooth nanowire. This shows that the complex growth process of constructing one-dimensional nanostructures from nanoparticles mainly involves the key steps of shape-oriented nanoparticle attachment, straightening, orientation correction, and mass redistribution, which provides an important mechanistic understanding for designing hierarchical nanomaterials and controlling nanocrystal self-assembly. In addition, heterostructured nanowires, as an important class of multifunctional nanomaterials, have attracted much attention for their growth process in solution. Niu *et al.* directly observed and tracked the growth trajectory of lead sulfide on gold nanorod seeds in liquid^177^ and found unique metal–semiconductor interface and heterostructure growth dynamics, including volume contraction of the core particles and interfacial strain-driven mass transport and new phase formation, which will help the future design and control of specific heterogeneous nanostructures.^177^ The development of *in situ* TEM has helped to explore new mechanisms of nanowire growth in solution. Cheek *et al.*^55^ discovered a novel electrochemical liquid–liquid–solid growth mechanism for Ge nanowires, where liquid metal nanodrops (gallium or indium) spontaneously react with dissolved GeO_2_ in the absence of an external power source, using secondary electrons from electron beam scattering to reduce GeO_2_ and facilitate Ge nanowire formation and growth on the liquid metal droplet surface. The growth process is affected by a number of factors, including the surface conditions of the liquid metal nanodrops, the size and density of the liquid metal nanodrops, the concentration of dissolved GeO_2_, and the intensity of the electron beam. It was observed in the study that the surface conditions of the liquid metal nanodrops were critical for the growth of the nanowires, and that proper ligand coverage was necessary for growth to occur, whereas excessive coverage of the surface ligands inhibited growth. In addition, the growth rate of Ge nanowires is limited by the rate of Ge supply to the crystal growth front rather than by the rate of crystallization at the liquid metal/solid Ge interface (Fig. 8B), suggesting that the growth process may be controlled by the rate of Ge supply in solution. The nanowire growth conditions away from thermodynamic equilibrium observed in the study provide a new way to control the morphology and crystallographic quality of the nanowires, suggesting that precise tuning of the nanowire structure can be achieved by regulating the growth conditions.

#### 3.3.3 Growth of 1D nanomaterials through gas–solid interactions

**Fig. 9:**
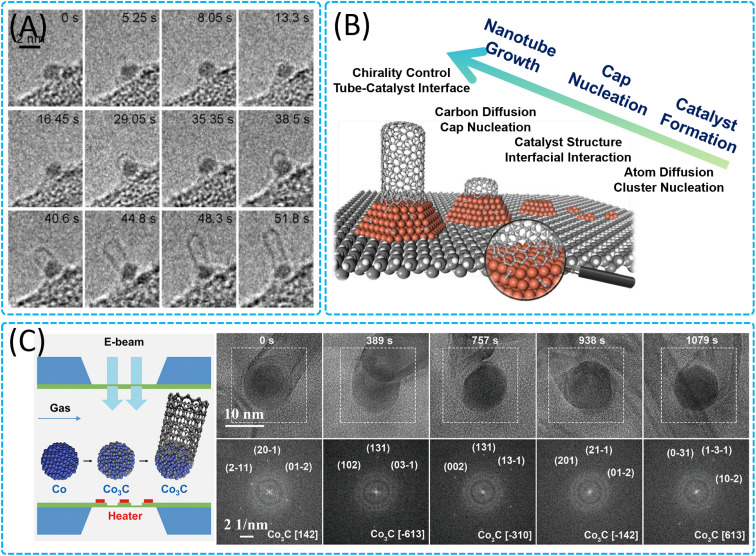
Catalyst-assisted 1D nanomaterial growth through gas–solid interactions. (A) Nucleation and growth process of a single-walled carbon nanotubes from a nanoparticle catalyst on a substrate. Reproduced with permission.^30^ Copyright 2008, American Chemical Society. (B) Schematic illustration of the catalyst evolution and growth process of single-walled carbon nanotubes as revealed by *in situ* ETEM. Reproduced with permission.^186^ Copyright 2022, American Chemical Society. (C) The phase evolution of cobalt catalyst nanoparticles during the incubation, nucleation, and growth stages of carbon nanotubes under near-atmospheric pressure using an *in situ* close-cell ETEM and phase structure of an active cobalt catalyst nanoparticle during carbon nanotube growth. Reproduced with permission.^31^ Copyright 2020, American Chemical Society.

The gas–solid reaction growth mechanism of 1D nanowires (gas–solid (VS),^178,179^ gas–solid–solid (VSS),^156,180^ catalyst-assisted gas–solid growth^180–183^ and autocatalytic growth mechanism^179,184^) involves the interaction of a precursor in the gas phase with a solid substrate or a catalyst at high temperatures, which ultimately forms nanowires (metal nanowires,^179,182,184^ semiconductor nanowires,^185^ carbon nanotubes (CNTs),^30,32,186–191^*etc.*). These mechanisms not only determine the growth rate, growth direction, diameter, length and crystal structure of nanowires, but also have a profound impact on the properties and applications of nanowires. For the study of the growth mechanism of metallic nanowires, researchers explored the growth of gold nanowires and platinum-based metal nanowires, where gold nanowires were synthesized by ultrasound-assisted synthesis on plasma-activated graphene templates, and platinum-based metal nanowires were prepared by hydrogen-assisted gas-phase synthesis without the presence of a catalyst. Ma *et al.* found that the growth of platinum-based metal nanowires belonged to the diffusion-assisted solid-state oriented attachment autocatalytic solid-state oriented adhesion of platinum-based metal nanowires.^179^ The growth of Pt-based metal nanowires was found to follow a diffusion-assisted autocatalytic growth mechanism of solid-state oriented attachment. This mechanism consists of four stages: the Pt precursors are reduced in a hydrogen atmosphere to form a large number of Pt nuclei, the Pt nuclei gradually grow to form Pt nanoparticles of a few nanometers by consuming smaller nuclei, the Pt nanoparticles are attached and fused to form Pt chains through specific orientation relationships, and these chains are further grown into ultrathin Pt nanowires. During the whole growth process, hydrogen plays a crucial role, which not only promotes the reduction of Pt precursors, but also changes the atomic diffusion rate on the Pt surface, especially on the {100} crystal surface, making the surface diffusion of Pt atoms more significant, thus promoting the formation of nanowires. In the absence of hydrogen, the Pt precursors only aggregated and failed to form nanowires. In addition, the researchers successfully synthesized Pt–Ni alloy nanowires with excellent electrocatalytic activity and stability, further demonstrating the universality and practicality of this growth mechanism and providing new insights into the structure-controlled synthesis of future metal nanowires. As an important class of one-dimensional nanomaterials, the growth mechanisms (apical growth mode and bottom growth mode) of CNTs directly affect the structural control, quality, and yield of CNTs, which in turn determines their potential applications in various fields. The researchers explored the dynamic behavior of different catalysts during the nucleation of carbon nanotubes by ETEM, including the diffusion of carbon atoms on the catalyst surface and the nucleation at the catalyst–CNT interface. It is shown that the growth of carbon nanotubes involves the diffusion of carbon atoms on the catalyst surface and the structural evolution. Among them, the growth mode of CNTs on catalysts is mainly the tip growth mode, in which the carbon source forms carbon atoms or carbon structures on the top of catalyst particles and gradually extends to form nanotubes. During the growth process, the catalyst particle serves as a template to promote the polymerization of carbon and the extension of the tube (Fig. 9A and B). Yoshida *et al.*^30^ found that for multi-walled carbon nanotubes (MWNTs), a graphene layer first forms on one face of the catalyst (Fe) nanoparticle, and then it gradually extends and bends along the face of the nanoparticle, and a new graphene layer nucleates underneath the existing layer (Fig. 9A), resulting in the nanoparticle's gradual deformation, eventually forming a protrusion from which carbon nanotubes grow rapidly. Moreover, Zhao *et al.* investigated that the physical state of the catalyst (solid or liquid) has a decisive influence on the diameter and chirality of single-walled carbon nanotubes during the growth process. The diameter of single-walled carbon nanotubes grown on solid catalysts (Co_7_W_6_) is smaller than that of the catalyst particles, whereas the diameter of single-walled carbon nanotubes grown on liquid catalysts (Co) is similar to that of the catalyst particles (Fig. 9B).^186^ Wang *et al.* explored another growth mode of carbon nanotubes (CNTs),^31^ the bottom-end growth mode, and investigated in detail the active phase of cobalt (Co) catalysts and the catalyst-assisted gas–solid growth mechanism during the growth of carbon nanotubes (Fig. 9C). The cobalt catalyst precursor was first deposited on a silicon nitride (SiN_*x*_) film, and metallic Co nanoparticles were formed by oxidation and reduction processes. During CNT growth, the active phase of the Co catalyst was the orthorhombic phase Co_3_C, and carbon atoms were supplied by diffusion through the surface and the nanoparticles–CNT interface rather than through the bulk phase of Co_3_C. They observed that the growth of CNTs followed a bottom-end growth pattern, where the growth of carbon nanotubes was guided by the bottom end connected to the catalyst particles. During the nucleation phase, the metal fcc Co nanoparticles rapidly transformed into orthorhombic phase Co_3_C nanoparticles, followed by the gradual formation of a graphite layer on the surface of the Co_3_C nanoparticles until the CNTs began to grow. The growth of the outer wall of MWCNTs is supplied by surface-diffused carbon atoms, while the growth of the inner wall may be realized by interfacial diffusion between the catalyst and CNTs. Thus, *in situ* TEM plays a key role in understanding the multiple growth mechanisms of metal-based, semiconductor-based and carbon-based nanowires and provides new ideas and insights for the structure-controlled synthesis of future nanowires.

### 3.4 Growth of 2D nanomaterials

2D nanomaterials are characterized by their ultrathin profiles and exhibit distinctive properties due to quantum confinement effects, which differentiate them from their bulk forms.^192–194^ These materials demonstrate remarkable potential in various high-tech applications, including next-generation electronics, optoelectronics, magnetism, spintronics, catalysis, and energy storage due to their unique geometric structures and extraordinary properties.^195–197^ The functionality of 2D nanomaterials is strongly related to their atomic structure, morphology, and the presence of defects and interfaces, which can be precisely engineered. Defects, which are common in both natural and synthesized crystals, can act as active sites, introducing localized electronic states and significantly enhancing the properties of the 2D materials. Interfaces between 2D materials and other components, as well as heterostructures created by stacking different 2D materials, can exhibit unique electronic and optoelectronic properties.^198^

*In situ* TEM has emerged as a cutting-edge technique for studying the growth mechanisms of 2D nanomaterials at the atomic scale.^199,200^ It provides real-time monitoring capabilities under various stimuli, including electron irradiation, thermal excitation, and voltage bias, offering unprecedented insights into nucleation, growth, and phase transformations.^19,201^ Advanced techniques such as aberration-corrected STEM and EELS have further enhanced the understanding of the structure–property relationships in 2D materials by enabling detailed examinations of their atomic structures, chemical compositions, and electronic properties.^46,202^ The current state of research leveraging *in situ* TEM showcases the ability to identify various polymorphs, defects, and interfaces in 2D nanomaterials. It also highlights the capacity for atom-by-atom chemical analysis and the study of excitons and phonons, which are crucial for understanding the properties of the 2D nanomaterials.

#### 3.4.1 Growth of 2D nanomaterials through solid-state interactions

In the field of exploring the synthesis of 2D materials, the high-temperature thermal decomposition method has garnered widespread attention due to its simplicity, scalability, and potential for precise control over growth conditions. This method utilizes the decomposition reaction of solid precursors under high-temperature conditions to generate 2D materials with specific structures and properties.^200,203^ However, variations in the interfacial migration rates^204^ and the presence of defects^205^ in the 2D materials during the high-temperature thermal decomposition process may affect the quality and performance of the materials. At the same time, although solid-phase high-temperature thermal decomposition reactions can grow 2D materials, understanding of the dynamic evolution and mechanisms during growth is still limited, which restricts precise control over material growth. The application of *in situ* heating sample holder technology in TEM provides a new perspective for the preparation and characterization study of 2D materials. It allows us to directly observe the growth process of 2D materials at the atomic scale, including nucleation, growth, and phase transitions of crystals, which is crucial for understanding the formation mechanisms of the materials. Additionally, by precisely controlling conditions such as heating rates and temperatures, it is possible to study the synthesis process of materials under simulated actual growth conditions, thereby achieving precise control over the structure and properties of the materials.

**Fig. 10:**
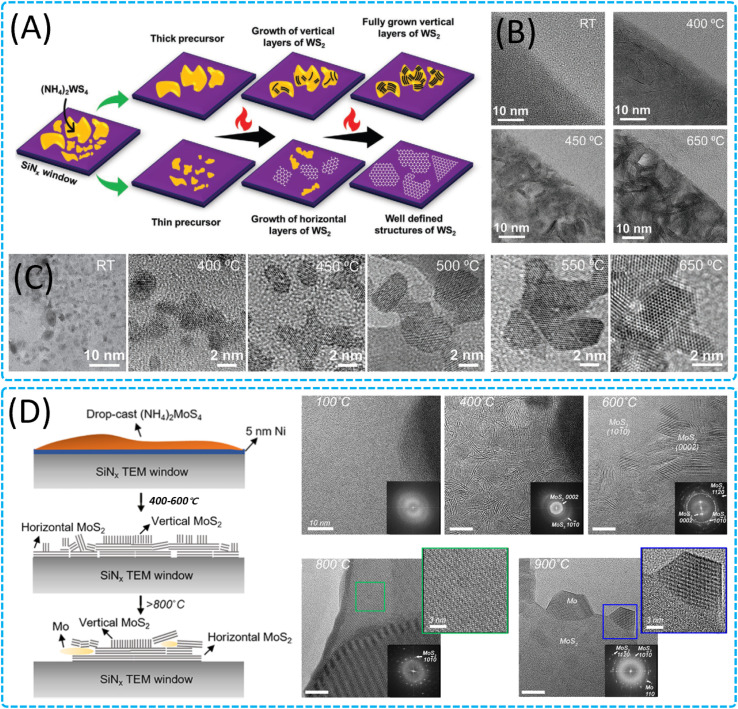
Structure evolution and growth of 2D nanomaterials through solid-state interactions. (A) Schematic illustration of the evolution of polycrystalline WS_2_ through thermolysis of an ammonium tetra-thiotungstate precursor, showing the growth of vertical layers and horizontal structures with heating in thick and thin precursor areas, respectively. (B) Growth of vertically aligned layers of WS_2_ at different temperatures. (C) Horizontal growth of WS_2_ layers at different temperatures.^206^ Copyright 2022, Wiley. (D) Illustration summarizing the formation of MoS_2_ crystals from the pure ammonium tetrathiomolybdate precursor and *in situ* TEM images showing the evolution of the ammonium tetrathiomolybdate precursor in the presence of a 5 nm Ni film during heating to different temperatures.^203^ Copyright 2019, American Chemical Society.

Researchers have utilized *in situ* TEM with an *in situ* heating holder to delve into the growth mechanisms of various 2D nanomaterials (WS_2_,^206^ MoS_2_,^49,51,203,204,207–209^ V_2_O_5_ (ref. 50), *etc.*) at high temperatures during solid-state reactions. The study reveals precise control over the vertical and horizontal growth of different 2D materials through the thermolysis of solid precursors, as well as multiple growth stages and various growth modes formed on different substrates. Gavhane *et al.* achieved control over the vertical and horizontal growth of WS_2_ by altering the thickness of the precursor (Fig. 10A–C),^206^ and the study found that on different metal-deposited heating chips, two layers of WS_2_ formed interference patterns by rotating at various angles relative to each other, providing a new perspective for understanding the growth dynamics of WS_2_. Additionally, Kondekar *et al.* discovered that a low concentration of Ni can significantly alter the crystallization and growth process of MoS_2_, leading to an increase in MoS_2_ crystal size, which may be due to changes in the migration rate of grain boundaries during the growth process (Fig. 10D).^203^ In contrast, a higher concentration of Ni inhibits the formation of MoS_2_, instead forming Ni and nickel sulfides. These findings indicate that the addition of other metal elements during synthesis plays a crucial role in the evolution of 2D nanomaterials. Regarding 2D V_2_O_5_ nanomaterials, Gavhane *et al.* utilized an *in situ* heating holder to observe in real time the formation process of two-dimensional V_2_O_5_ nanostructures, including the growth of orthogonal V_2_O_5_ 2D nanosheets and 1D nanobelts.^50^ The study also revealed the phase transition process of V_2_O_5_ to VO_2_ and optimized the temperature range required for the growth of V_2_O_5_ nanostructures. These studies provide in-depth insights into the growth dynamics of WS_2_, MoS_2_, and V_2_O_5_ and offer effective pathways for the preparation of 2D nanomaterials. Furthermore, Kotakoski *et al.* employed *in situ* STEM combined with a deep learning framework to explore the dynamic process of MoS_2_ restructuring from 2D to 3D configurations and its growth on graphene.^209^ These studies not only provide an in-depth atomic-level understanding of the growth and structure of 2D nanomaterials but also demonstrate the potential of deep learning technology in 2D material research, offering new avenues for exploring novel structures and properties. Concurrently, the study investigated the dynamic behavior and structural changes of nanocrystalline graphene under high-temperature conditions, as well as the structural evolution of vertically aligned 2D MoS_2_ layers, providing key insights into the structural stability of general van der Waals 2D crystals and offering valuable technical guidance for material design and optimization. Overall, the results from *in situ* heating studies on pure solid-state reactions are crucial for guiding the synthesis, manufacturing, and customization of functional characteristics of 2D nanomaterials, providing new understanding for the controlled synthesis of large-area 2D nanomaterials, and holding the potential for achieving atomic-level precise control and growth of 2D nanomaterials during solid-state reactions.

#### 3.4.2 Growth of 2D nanomaterials in the liquid phase

**Fig. 11:**
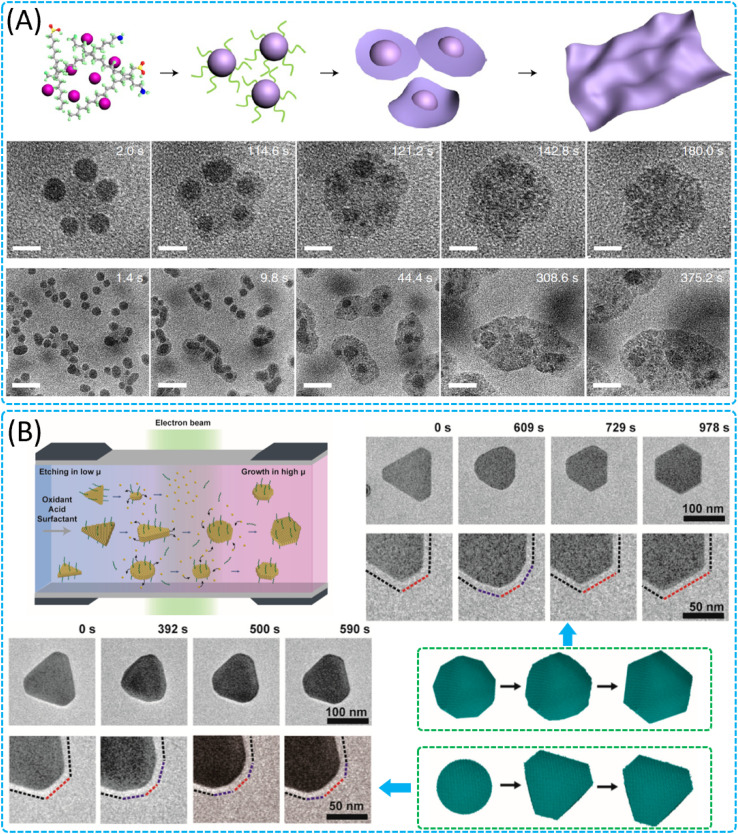
Growth and shape transformation of 2D nanomaterials in the liquid phase. (A) Schematic showing the formation of a 2D nanosheet from a molecular precursor solution with the pathway of 3D nanoparticle growth and subsequent 3D-to-2D transformations. Sequential images show a few cobalt nickel oxide nanoparticles transforming into a 2D nanosheet and the formation of cobalt nickel oxide nanosheets through the growth of 3D nanoparticles and 3D-to-2D transformations. Reproduced with permission.^215^ Copyright 2019, Springer Nature. (B) Schematic illustration of an LPTEM cell for observing diverse shape transformations of Au nanocrystals. Reproduced with permission.^226^ Copyright 2023, American Chemical Society.

Liquid-phase preparation methods allow precise control of the size, morphology and structure of 2D nanomaterials at the molecular level, enabling fine-tuning of the electronic, optical and catalytic properties of 2D materials.^210^ The development of *in situ* liquid-cell TEM technology has enabled real-time imaging of the reaction process in a liquid environment with high spatial and temporal resolution, providing a new perspective on molecular-scale dynamics and a deeper understanding of the growth mechanisms and morphological evolution of materials, thus optimising synthesis conditions and improving material quality and yield. These *in situ* studies mainly include the growth mechanisms of 2D nanomaterials (metal monolithic 2D materials,^211–213^ 2D transition metal oxides^214–216^ and sulfur compounds,^215^ core–shell 2D materials,^217^ core–shell structures,^218,219^ MOF-based 2D structures.^220–223^*etc.*), the kinetic pathways during crystal formation, and the superstructure assembly of 2D materials.^223,224^ The formation mechanism of conventional 2D materials such as graphene, metal hydroxides^225^ or sulfur compounds is relatively well defined due to their inherent layered atomic structure. However, the mechanism of how non-lamellar crystals form 2D nanosheets is not clear. Zheng's group thoroughly investigated the formation process of non-lamellar transition metal oxide nanosheets and revealed the growth mechanism of how non-lamellar crystals form 2D nanosheets (Fig. 11A).^215^ Experimentally, it was observed that under certain conditions, 3D nanoparticles start to grow as the reaction time progresses. During the transition, specific crystal planes grow at different rates, resulting in nanoparticles with specific morphologies, such as ‘butterfly’ shapes or squares. Competition between surface and edge energies, size effects, and ligand–surface interactions are the main factors leading to the transition from 3D nanoparticles to 2D nanosheets.

The structure–property relationship of noble metal nanocrystals is crucial for their applications in various fields such as catalysis and sensing. Noble metal nanosheets exhibit unique behaviours during shape transformation due to their high surface-to-volume ratio and dynamic surface reactivity, which include adsorption, desorption, and diffusion of surface atoms, processes that are critical for the overall shape change. Therefore, researchers have delved into the growth kinetics and formation mechanisms of gold nanosheets,^212,213,227^ silver nanosheets,^228^ and palladium dendritic nanosheets.^211^ Alloyeau *et al.* found that the growth of colloidal nanoparticles is affected by a combination of kinetic and thermodynamic effects and that by controlling the electron dosage, it is possible to control the growth rate directly, thus quantifying the influence of kinetic effects on planar nanoparticle formation.^227^ Park *et al.* revealed that at lower electron doses, the growth of gold nanosheets is driven by thermodynamics, and the formation and shape of nanosheets are directly related to the formation of twinned surfaces during growth.^212^ Jin *et al.* also found that the growth rate of Au nanocrystals can be precisely controlled by adjusting the solution chemistry, in particular pH and chloride ion concentration, which is important for the design and synthesis of nanostructures with specific shapes and structures.^213^ In addition, Choi *et al.*^226^ explored the shape change mechanism of Au nanosheets (Fig. 11B–E) and found that the diffusion of nanocrystal surface atoms is the main determinant of the final structure in the shape change and that this rapid diffusion of surface atoms leads to a truncated morphology transition of unstable crystal surfaces, thus minimising the surface energy. Liquid *in situ* experiments revealed that oxidative etching of gold nanoprisms and subsequent structural remodelling of the crystal faces were induced by changing the chemical potential in the reaction solution and that diffusion of surface atoms on the exposed crystal faces led to the development of unstable {220} crystal faces into stable {111} crystal faces, resulting in truncated morphologies with minimal surface energy. This finding not only provides a new perspective for understanding the formation mechanism of nanocrystals of various shapes, but also has important implications for the controlled synthesis of colloidal nanocrystals. Meanwhile, for silver nanosheets, E studies revealed a dissolution-re-growth mechanism from triangular to hexagonal shapes, providing a potential pathway for the synthesis of Ag HNPs with controllable shapes and sizes. These findings not only deepen the understanding of the microscopic formation process of nanosheets, but also elucidate the origin of the observed reversible shape changes, providing new insights into the rational design of controllable nanocrystal shapes in the future, as well as key prerequisites for the understanding of the growth mechanism of nanomaterials and the control of shape-dependent properties.

#### 3.4.3 Growth of 2D nanomaterials through gas–solid interactions

**Fig. 12:**
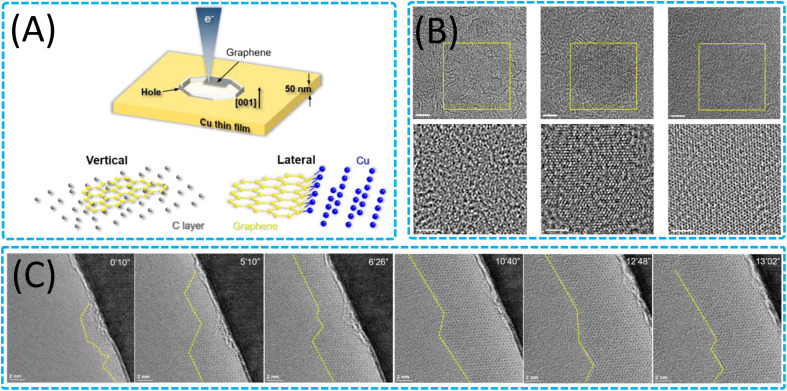
Growth of 2D nanomaterials through gas–solid interactions. (A) ETEM experimental setup and typical observations of *in situ* graphene growth. (B) Nucleation and growth of graphene from an amorphous C layer. (C) Time-resolved HRTEM images showing the lateral epitaxial growth of graphene on the Cu edge. Reproduced with permission.^230^ Copyright 2020, American Chemical Society.

Chemical vapour deposition can achieve high-quality, uniform and controllable growth of 2D nanomaterials on large-area substrates and is a key synthetic method for the preparation of 2D nanomaterials. However, the deposition mechanism of different atoms during the synthesis process and the gas–solid growth mechanism of 2D layered nanomaterials still need to be explored and investigated in depth.^229^ Among them, graphene, as a 2D material with high carrier mobility at room temperature, is considered to be one of the most promising candidates for next-generation electronic devices. Researchers investigated the growth mechanism of graphene on different substrates (Cu,^230^ Ni,^231^ and SiC^232^) by *in situ* TEM. Liu *et al.* used aberration-corrected ambient TEM (AC-ETEM) to observe in real time the nucleation and growth of graphene on an atomic scale Cu substrate (Fig. 12A),^230^ and the nucleation of graphene nuclei from the amorphous was observed under a CO/C_2_H_4_ atmosphere, carbon atoms for nucleation and growth with gradual ordering of in-plane carbon atoms (Fig. 12B). In addition, the growth of graphene on the edge of copper has a unique lateral epitaxial growth process as well as a step-flow process under a CO/CO_2_ atmosphere (Fig. 12C). Kling *et al.*^231^ found that during the growth of layered carbon structures on nickel (Ni) substrates using acetylene as a carbon precursor, the growth of graphene occurred in two phases by observing the growth of individual graphene layers on the Ni surface, with an initial fast growth phase that was not strongly pressure dependent, followed by a much slower growth phase that was strongly pressure dependent. Meanwhile, Yu *et al.*^232^ observed the growth of graphene on SiC surfaces by combining *in situ* TEM at 1000 °C at the atomic scale. The results show the sequential decomposition of three SiC layers to form graphene. Sublimation of the first layer leads to the formation of carbon clusters containing short chains and hexagonal rings, and these can be considered as the core of graphene growth. The decomposition of the second layer leads to the joining of new chains with already formed clusters to form a network with large pores. Eventually, the release of carbon atoms from the third layer leads to the disappearance of chains and macropores from the network, forming a complete graphene layer. This study provides a clear picture for understanding the epitaxial growth mechanism of monolayer graphene on SiC. These atomic-scale real-time observations and analyses provide direct evidence for understanding the growth mechanism of graphene and can be extended to other 2D materials.

## 4 Electron-beam induced synthesis for nanomaterials

Electron beams play a pivotal role in the synthesis of nanostructures, offering a versatile and precise method for manipulating materials at the nanoscale.^233–236^ The high energy of electron beams allows them to induce a range of physical and chemical changes in materials, such as knock-on displacement,^237^ sputtering,^238^ and radiolysis,^239^ which are essential for nanostructure synthesis. These interactions can lead to the formation of nanoparticles,^233,240^ nanowires,^241^ nanosheets,^234,242,243^ and more complex geometries, all through *in situ* processes within TEM. One significant advantage of using electron beams for nanostructure synthesis is the ability to control the process with atomic precision, which can directly affect the final properties of the nanostructures. TEM provides real-time temporal resolution, enabling researchers to observe the microstructural evolution as nanostructures are synthesized. On the other hand, electron-beam-induced synthesis can be conducted without specialized specimen holders or peripheral equipment, making it a simpler and rapidly growing approach. It discusses various protocols for synthesizing different dimensional nanostructures, including 0D nanoparticles,^235^ 1D nanowires/nanotubes,^244^ 2D films,^238^ and other exotic geometries like nano-trees or nano-dendrites.^245^

**Fig. 13:**
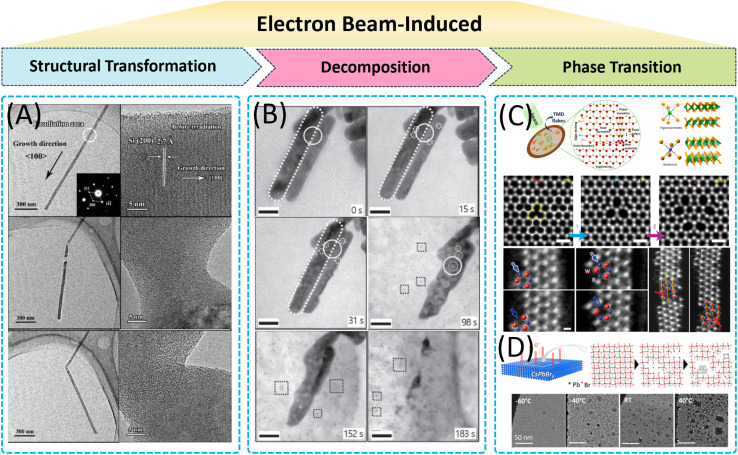
Electron-beam induced synthesis of nanomaterials: electron-beam-induced (A) *in situ* structural transformation in 1D nanomaterials. Reproduced with permission.^244^ Copyright 2015, Science China Press. (B) Decomposition of Bi_2_S_3_ nanorods in water. Reproduced with permission.^246^ Copyright 2021, IOP. Phase transition of (C) 2D transition metal dichalcogenides. Reproduced with permission.^46^ Copyright 2019, American Chemical Society. (D) Colloidal cesium lead halide perovskite nanocrystals. Reproduced with permission.^249^ Copyright 2017, American Chemical Society.

There has been a lot of research focus on the investigation of nanomaterials and their transformations under electron beam irradiation, utilizing advanced TEM techniques. These studies explore the structural changes,^234,243^ decomposition,^239,246^ and phase transitions^247–250^ processes of nanomaterials with different morphologies such as 0D, 1D and 2D materials. Zhu *et al.* investigated the effects of electron-beam irradiation on the structural transformation of silicon (Si) and zinc oxide (ZnO) nanowires. They revealed that electron-beam irradiation can induce a crystal-to-amorphous transition in Si nanowires and surface reconstruction in ZnO nanowires (Fig. 13A).^244^ These transformations demonstrate the potential for localized modification of one-dimensional nanomaterials using electron beams. Kim *et al.* observed the rapid decomposition of Bi_2_S_3_ under electron beam irradiation in water (Fig. 13B),^246^ providing insights into the stability and potential applications of such photocatalysts in addressing energy and environmental issues. Meanwhile, more and more researchers are focusing on the transformative effects of electron beam irradiation on nanomaterials, specifically on transition metal dichalcogenides (Fig. 13C)^46^ and cesium lead halide perovskites (Fig. 13D).^249^ Mendes *et al.*^46^ and Manna *et al.*^249^ employed TEM to investigate the structural and compositional changes induced by electron irradiation. They explored how electron beams can stimulate desorption of atoms, induce phase transformations, and trigger the nucleation and growth of nanoparticles within these materials. The research underscored the potential of electron beam manipulation for material property tuning and the development of nanodevices, while also highlighting the challenges associated with controlling the electron irradiation-induced processes for stable material synthesis and applications in optoelectronics and photovoltaics.

Furthermore, the electron-beam specimen interactions are crucial for understanding the physical background behind the growth mechanisms. The section also discusses the challenges and limitations of using electron beams, such as specimen charging and the risk of inducing uncontrollable structural transformations. The electron-beam-induced synthesis of nanostructures is a powerful technique that leverages the precise interactions between electron beams and materials to create nanostructures with tailored properties.^248^ This method is particularly valuable for research and development in nanotechnology, offering a platform for innovation and the potential for large-scale industrial applications once the challenges are addressed.^249^

## 5 Challenges and conclusion

*In situ* TEM has proven to be an invaluable tool for investigating the synthesis of nanomaterials at the atomic scale, offering unprecedented insights into nucleation, growth, and structural transformations. However, despite its substantial contributions to the field, there are several challenges associated with *in situ* TEM that need to be addressed to fully harness its potential in nanomaterial synthesis.

### 5.1 Integration with other techniques

**Fig. 14:**
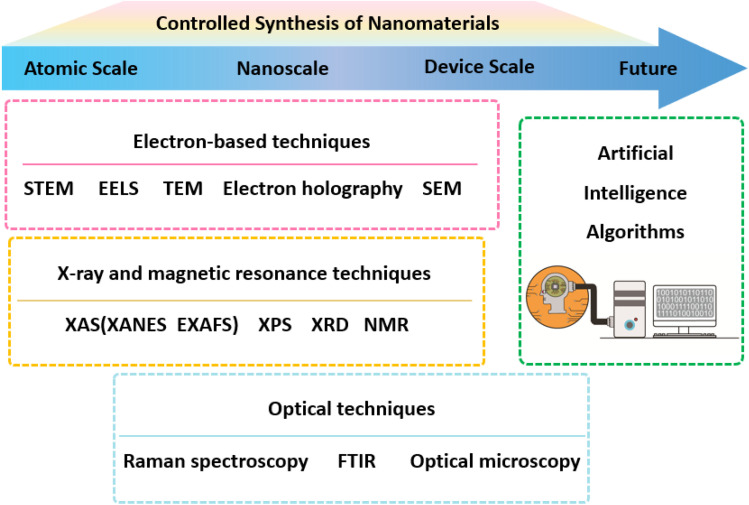
The most important *in situ* characterization techniques with their spatial resolution scales and the corresponding detection targets in the synthesis of nanomaterials.

Although *in situ* TEM can provide structural information, real-time chemical characterization at the atomic scale during synthesis is still limited. Firstly, combining *in situ* TEM with advanced spectroscopic methods for real-time chemical analysis is an area that requires further development. Therefore, to gain a comprehensive understanding of nanomaterial synthesis, *in situ* TEM needs to be integrated with other characterization techniques such as spectroscopy, diffraction, and tomography. Secondly, although *in situ* TEM excels at providing vivid, real-time images of nanomaterials' structural and chemical transformations within their chemical milieus, a complete grasp of their synthetic intricacies demands a multifaceted investigational approach. To counter this, an array of innovative *in situ* characterization techniques has swiftly come to the forefront, including but not limited to *in situ* Raman spectroscopy,^251–253^*in situ* infrared spectroscopy,^254–256^*in situ* X-ray diffraction (XRD),^257–259^*in situ* nuclear magnetic resonance (NMR),^260–263^*in situ* X-ray photoelectron spectroscopy (XPS),^264–266^ and *in situ* X-ray absorption fine structure (XAFS).^267,268^ These advanced techniques have filled the gaps left by conventional approaches. Moreover, the harmonization of these cutting-edge *in situ* methods with established characterization tools is forging new avenues for the invention of novel analytical methodologies. This integrated approach is set to facilitate a more rounded appreciation of nanomaterial synthesis mechanisms, thereby enhancing our ability to design and fabricate materials with tailored properties for specific applications (Fig. 14).^269^

### 5.2 Complex reaction environments

Replicating realistic synthesis conditions within the high-vacuum environment of a TEM remains a challenge. The need to integrate multiple external stimuli, such as liquid, gas, heat, and light, into a single *in situ* TEM experiment is crucial for mimicking real-world synthesis processes. On the one hand, it is difficult to maintain precise control of the gas and liquid environments within the TEM, including pressure and composition. The spatial and temporal resolution can be compromised due to gas scattering and the need for high-pressure compatibility. On the other hand, accurate temperature measurement at the nanoscale is challenging, particularly when considering the heat effect of the electron beam. This can affect the actual phase transformation temperature and dynamics.

### 5.3 Data acquisition and analysis

The acquisition of high-quality, high-resolution data in a timely manner is essential for understanding complex nanomaterial synthesis processes. However, the current limitations in data acquisition systems, such as frame rates and image quality, can hinder the detailed analysis of dynamic processes. In addition, understanding the role of interfaces and compositional changes during nanomaterial synthesis is critical. However, current *in situ* TEM techniques may struggle to provide detailed information on the chemical state, valence, and distribution of elements, particularly light elements, which are often involved in catalytic processes. Hence, the interpretation of *in situ* TEM observations can be complex, particularly when distinguishing between different growth mechanisms or understanding the influence of various reaction parameters. Developing a comprehensive understanding that links observations to underlying mechanisms is an ongoing challenge.

### 5.4 Temporal and spatial resolution

Capturing the dynamics of nanomaterial synthesis requires high temporal resolution to follow fast processes and high spatial resolution to observe atomic-scale changes. Current *in situ* TEM techniques may not always provide the necessary resolution to capture all relevant details, particularly for very fast or small-scale phenomena.

### 5.5 Electron beam interaction

The electron beam used in TEM can interact with the sample, causing effects such as heating, knock-on damage, or charging. These interactions can alter the sample's structure and chemistry, potentially leading to observations that do not accurately represent the undisturbed synthesis process.

### 5.6 Sample preparation and stability

Preparing samples that are representative of actual synthesis conditions and maintaining their stability under electron beams are non-trivial tasks. The need for specialized holders and the potential for sample contamination or damage during preparation and observation add layers of complexity. Meanwhile, achieving stable loading of nanomaterials within the TEM and maintaining the sample under test conditions without drift is a significant challenge, especially for quantitative nanomechanical tests that require precision.

As mentioned above, the *in situ* TEM method is essential for obtaining high-resolution data on nanocrystal growth in relation to space, time, and energy. We anticipate the development of more intricate *in situ* cultivation settings and a variety of experimental approaches within TEM, including hydrothermal and CVD techniques. Furthermore, the integration of cutting-edge characterization methodologies and advanced data analytics, such as high-throughput experimentation and artificial intelligence algorithms, is anticipated. This synergy will facilitate a more profound comprehension of the underlying nucleation and growth mechanisms of nanocrystals, enabling the meticulous design and crafting of nanocrystals tailored with specific structural and functional attributes. By leveraging these advanced techniques, researchers will gain the ability to elucidate the intricate dynamics of nanocrystal formation with unprecedented clarity, leading to advancements in the precise engineering of materials with customized properties for a wide array of applications. These advances hold immense promise for a wide range of applications in nanomaterials.

## Data availability

No primary research results, software or code have been included and no new data were generated or analysed as part of this review.

## Author contributions

All the authors contributed to the literature search, writing, and editing of this review.

## Conflicts of interest

The authors declare no conflict of interest.
